# What Links an Increased Cardiovascular Risk and Inflammatory Bowel Disease? A Narrative Review

**DOI:** 10.3390/nu13082661

**Published:** 2021-07-30

**Authors:** Liliana Łykowska-Szuber, Anna Maria Rychter, Magdalena Dudek, Alicja Ewa Ratajczak, Aleksandra Szymczak-Tomczak, Agnieszka Zawada, Piotr Eder, Maciej Lesiak, Agnieszka Dobrowolska, Iwona Krela-Kaźmierczak

**Affiliations:** 1 Department of Gastroenterology, Dietetics and Internal Diseases, Poznan University of Medical Sciences, 60-355 Poznan, Poland; lszuber@wp.pl (L.Ł.-S.); alicjaewaratajczak@gmail.com (A.E.R.); aleksandra.szymczak@o2.pl (A.S.-T.); aga.zawada@gmail.com (A.Z.); piotr.eder@op.pl (P.E.); agdob@ump.edu.pl (A.D.); krela@op.pl (I.K.-K.); 2 1st Department of Cardiology, Poznan University of Medical Sciences, 61-848 Poznan, Poland; magdalena.dudek@skpp.edu.pl (M.D.); maciej.lesiak@skpp.edu.pl (M.L.)

**Keywords:** cardiovascular disease, inflammatory bowel disease, diet, dysbiosis, pharmacotherapy

## Abstract

Several studies have shown increased rates of cardiovascular disease (CVD) in patients suffering from inflammatory bowel disease (IBD), particularly in cases of early atherosclerosis and myocardial infarction. IBD most frequently begins at an early age, patients usually present normal weight and remain under constant care of a physician, as well as of a nutritionist. Therefore, the classical risk factors of CVD are not reflected in the higher prevalence of CVD in the IBD population. Still, both groups are characterised by chronic inflammation and display similar physiopathological mechanisms. In the course of IBD, increased concentrations of pro-inflammatory cytokines, such as C-reactive protein (CRP) and homocysteine, may lead to endothelial dysfunctions and the development of CVD. Furthermore, gut microbiota dysbiosis in patients with IBD also constitutes a risk factor for an increased susceptibility to cardiovascular disease and atherosclerosis. Additionally, diet is an essential factor affecting both positively and negatively the course of the aforementioned diseases, whereas several dietary patterns may also influence the association between IBD and CVD. Thus, it is essential to investigate the factors responsible for the increased cardiovascular (CV) risk in this group of patients. Our paper attempts to review the role of potential inflammatory and nutritional factors, as well as intestinal dysbiosis and pharmacotherapy, in the increased risk of CVD in IBD patients.

## 1. Introduction

According to recent studies, patients with inflammatory bowel disease (IBD) present an increased risk of developing cardiovascular diseases (CVD); however, there is relatively little literature data regarding the relationship between IBD and CVD. Therefore, these observations are of great interest to researchers, and simultaneously they open up a field for discussion [1,2].

Cardiovascular diseases—disorders of the heart and the circulatory system—are the leading global cause of mortality and morbidity [3]. According to the 2019 report from the European Society of Cardiology (ESC), there are more than 11 million new cases of CVD across Europe every year [4]. Moreover, CVD causes more than 4 million deaths in the same region, and is the most significant cause of premature mortality and Disability-Adjusted Life Years (“DALYS”) in Europe [5]. On the basis of epidemiological and medical studies, researchers managed to identify numerous risk factors and causes of CVD some of which are invariable, e.g., age, gender or genetic heritage; whereas others are variable, including smoking tobacco, physical inactivity, poor eating habits, type 2 diabetes, dyslipidaemia, elevated blood pressure, or obesity [6]. Nevertheless, the main CVD risk factors comprise smoking, alcohol consumption and mean levels of blood cholesterol, although the prevalence of overweight/obesity and diabetes is increasing [5]. In fact, more than 9 million premature deaths from CVD worldwide can be attributed to poor eating habits, which accounts for 2% of all CVD-related deaths in 2016 [7]. Hence, CVD is increasingly becoming a diet-dependent disease, which affects the population of young people, amongst whom a particular group of patients with IBD can be distinguished. It has been established that there is an increased risk of cardiovascular events in the inflammatory autoimmune disorders, such as IBD [8,9,10,11]. Atherosclerosis and inflammatory bowel disease are often considered as two separate entities, despite the fact that atherosclerosis is associated with the dysregulation of immune systems along with platelet and endothelial dysfunction [12,13].

In the general population, the occurrence of risk factors correlates with a higher risk of cardiovascular events [14,15,16,17]. However, IBD patients present with higher rates of atherosclerotic cardiovascular disease (ASCVD) in spite of lower rates of the abovementioned risk factors [18]. Several potential mechanisms have been suggested to account for the increased cardiovascular risk in patients with IBD, the most crucial of which is the underlying pro-inflammatory state acting as a strong stimulus for endothelial dysfunction and thrombogenesis [19,20]. Common pro-inflammatory markers, such as C-reactive protein (CRP) and homocysteine, are known to be elevated in patients with CVD. What is more, they are also markers of chronic systemic inflammation in conditions, such as Systemic Lupus Erythematosus (SLE), rheumatoid arthritis and IBD [21,22,23]. The mechanisms leading to endothelial dysfunction in IBD include chronically increased levels of pro-inflammatory cytokines (CRP, IL-6, TNF-α and oxidative stress [24,25]. Superoxide anion and other reactive oxygen species (ROS) are able to stimulate nuclear factor kappa B (NF-kB), which activates a variety of pro-inflammatory cytokines, including tumour necrosis factor-alpha (TNF-α) and interleukin-1 [26]. In turn, TNF- α increases inflammation in endothelial cells by increasing cell adhesion molecule expression [26]. Additionally, ROS induction increases the expression of ICAM-1 (intracellular adhesion molecule 1), MCP-1 (monocyte reactant protein 1), E-selectin, and macrophage infiltration [27]. This mechanism leads to endothelial cell apoptosis, micro- and macrovascular dysfunction, as well as decreases endothelial-dependent vasodilatation and increases pro-thrombotic state.

Moreover, IBD patients are at increased risk of early atherosclerosis and myocardial infarction [8,28,29]. According to a study conducted in 2014 by Aggarwal et al., patients with IBD presented an earlier onset of CVD than non-IBD patients [30]. As pointed out in one of the recent studies involving 29 million patients, the prevalence of acute myocardial infarction (AMI) was higher in both patients with ulcerative colitis (UC) and Crohn’s disease (CD), as compared to non-IBD patients, whereas the highest risk of AMI was observed in the group of young female patients with IBD aged 30–34 [2]. In fact, the studies suggest that an active disease is associated with an increased risk of cardiovascular (CV) events. The study from Denmark demonstrated that the risk of AMI, stroke and CV death was significantly higher in the course of exacerbations of IBD, although it was similar to the control group during remission [8]. What is more, IBD patients have a 2–3-fold higher risk of venous thromboembolism than the general population, especially during flares [31]. Patients from this group have a predisposition for atrial and ventricular arrhythmias and conduction disturbances [32]. In their meta-analysis, Zuin et al. reported an increased risk of atrial fibrillation in BD patients in comparison to the general population [33]. Similar conclusions were found in a retrospective analysis of IBD cohorts done by Pattanshetty et al. [34]. Possible cardiovascular manifestations of IBD, mostly immune-related consequences, comprise pericarditis, myocarditis, venous and arterial thromboembolism, left ventricle impairment, arrhythmias and conduction disorders, infective endocarditis, valvulopathy, and Takayasu arteritis [35]. All the cardiovascular manifestations of IBD mentioned previously, as well as a chronic inflammatory status associated with IBD may lead to myocardial fibrosis, which finally results in left ventricle (LV) impairment and chronic heart failure [35]. Therefore, strategies of control and reduction of cardiovascular risk should be implemented in this particular group of patients.

Considering an increasing incidence of IBD and the observed complications of CVD, which can contribute to a higher mortality, it is necessary to identify the factors responsible for an increased CV risk in this group of patients. This paper attempts to review the role of the potential inflammatory and nutritional factors in IBD patients likely to increase the risk of CVD. The keywords that were used include “inflammatory bowel disease”, “cardiovascular disease”, “Mediterranean diet”, “Western diet”, “inflammation”, “pharmacotherapy”, “glucocorticoids”, “intestinal microbiota”, “intestinal dysbiosis”.

## 2. Cardiovascular Risk in Patients with IBD

### 2.1. IBD and Ischaemic Heart Disease

The aetiology of IBD has not been fully understood; nevertheless, it has been suggested that inflammation is one of the most essential and crucial factors. Although more attention is being paid to the association between CVD and IBD, several aspects remain uncertain and the results are inconsistent. A growing body of research suggests that IBD patients have an increased risk of ischaemic heart disease (IHA) and myocardial infarction (MI) [10], while several large studies indicate that both Crohn’s disease (CD) and ulcerative colitis (UC) patients present an increased risk of CVD. A large population study conducted in the United States of America in 2019 revealed that the risk of MI was higher among patients with IBD when compared to non-IBD individuals: the frequency of MI-UC 6.7% vs. CD 8.8% vs. non-IBD 3.3%, odds ratio [OR] for UC 2.09 [2.04–2.13] and CD 2.79 [2.74–2.85]. Moreover, data regarding gender show that this risk is higher among women with IBD than in men with IBD, particularly in younger individuals, reaching its peak between 30 and 34 years of age. However, the reasons for this phenomenon have not been fully understood. Possibly, the use of contraceptive pills and higher CRP concentrations among women may be crucial [2,9,36].

Simultaneously, several studies have suggested that the risk of acute cardiac injury mortality is lower among patients with IBD when compared with non-IBD patients. It could be explained by the fact that patients with IBD may respond to the pro-inflammatory cytokines released during cardiac arrest due to the chronic inflammation associated with the underlying disease [37].

In 2021 Preettika Sinh et al. published an interesting study which aimed at investigating the outcomes of myocardial infarction in 2,629,161 patients, including 3607 with UC and 3784 with CD. It demonstrated that IBD did not impact in-hospital mortality due to MI. Nevertheless, patients with MI with IBD were hospitalised for a longer period of time, whereas UC accounted for higher hospitalisation costs [38].

Many studies confirmed an increased risk of ASCVD in IBD patients when compared with the general population. Pemmasani et al. published a study in which they analysed differences in the risk profiles, treatment strategies and in-hospital mortality between patients with acute coronary syndromes (ACS) and IBD (24,220 patients) and ACS without IBD (6,872,415 patients). The results are particularly significant, as they demonstrated that patients with IBD had a lower baseline incidence of cardiovascular risk factors. Moreover, they showed that in-hospital mortality was lower in patients with IBD compared to the in-hospital group. (3.9% vs. 5.3%). On the other hand, the risk factors, ACS and mortality strategies for CD and CU were comparable. Nevertheless, it should be noted that coagulopathy, weight loss, gastrointestinal bleeding were more common in patients with IBD and constituted an additional predictor of mortality [39].

British researchers obtained similar results. They conducted a cohort analysis of the association between IBD, disease activity and the risk of myocardial infarction, stroke and cardiovascular death. Although they did not find a significant increase in vascular events in patients with IBD in general, the study demonstrated that the incidence of the events correlated with a higher disease activity [40].

Finally, the study of Kobo O. et al. assessed the complications following the supercutaneous coronary intervention in IBD patients. This study showed that IBD was associated with a lower risk of in-hospital complications following percutaneous coronary intervention (PCI) other than severe haemorrhage [41], which indicates that appropriate and effective treatment of IBD may be the basis for reducing the risk of ACS and reducing complications after PCI.

### 2.2. IBD and Venous Thromboembolic Events

Chronic inflammation in IBD leads to the activation of pro-coagulant mechanisms. Moreover, thrombocythaemia is also frequently observed among this population, as the aforementioned conditions may increase the rates of thromboembolic events [42].

#### 2.2.1. Venous Thromboembolism Incidents in IBD

It has been observed that in the IBD population, the risk of venous thromboembolism incidents increases, and this association has been widely studied. On the basis of literature data, patients with IBD presented an increased risk of pulmonary embolism, deep-vein thrombosis, as well as portal vein thrombosis when compared with the general population [28].

#### 2.2.2. Arterial Thromboembolism and Cerebrovascular Events in IBD

Currently, there is no reliable evidence that IBD increases the risk of arterial thromboembolism events, since the available data are inconclusive and stem from retrospective studies. However, an interesting meta-analysis was conducted by researchers from Minnesota, in which they showed that in patients with IBD, the risk of cardiovascular events increases, and it is gender-specific (it increases among women over 40 years of age) [9].

## 3. Risk Factors for CVD in IBD Patients

### 3.1. Inflammation

The role of pro-inflammatory cytokines in the pathogenesis of CVD is well documented. Many studies proved that several inflammatory markers—such as CRP, TNF-α, interleukin (IL)-6, IL-1β—play an essential role in the atherosclerotic process and increased odds of CV events [43]. Zhao et al. 7.5 years long study on 7600 patients with triple-vessel coronary disease presented interesting results; the leucocytes level was an independent risk factor for mortality and severe CV events [44]. As mentioned above, chronic inflammation—characteristic for IBD and associated with increased concentrations of pro-inflammatory cytokines—is the essential factor associated with the severity of IBD. Therefore, it seems essential to address the question whether the chronic inflammation in IBD also negatively affects the endothelium. TNF-α–pro-atherothrombotic cytokine, is increased in both UC and CD and increases the expression of VCAM-1 (vascular cell adhesion protein-1), which in turn contributes to the interaction of leukocytes with endothelium [45,46]. It should be remembered that endothelial dysfunction is also involved in the pathogenesis of IBD. Moreover, Danes et al. observed that the use of infliximab, blocking the TNF-α, also decreases the expression of VCAM-1 from the intestinal mucosa from intestinal micro veins, which further inhibits their inflammation and interaction with the T-cells [47]. Further, one of the consequences of chronic inflammation among IBD patients is aortic stiffening; however, as Zanoli et al. have shown, long-term anti-TNFα therapy reduces aortic pulse-wave velocity in IBD population, which suggests that inflammation therapy may reduce CV risk in IBD patients [48].

Vascular endothelial growth factor (VEGF)—mediating angiogenesis—is another cytokine which increases inflammation in the intestines [49]. Interestingly, it is a pro-atherogenic factor at the same time. However, its protective influence, associated with the stimulation of new veins of the collateral circulation, may also be found in the literature [50].

CRP is the most frequently used inflammatory biomarker both in the clinical practice and in the literature. It has been established that its concentration increases in an active phase of IBD, thus proving the presence of inflammation. Furthermore, the CV risk is higher when CRP concentrations are increased. Nevertheless, according to the new studies, the longer the exposure, the higher the risk, which in turn could suggest that the time of increased CRP concentrations is more critical than the CRP levels. This observation may be particularly essential in the IBD population presenting with chronic inflammation [51].

### 3.2. Drugs Used in IBD and CV Risk

The most frequently used drugs in IBD are 5-aminosalicylates, glucocorticoids (GCs), immunomodulatory drugs, and biological drugs. Although GCs are used as a basic treatment of inflammation in IBD, they also clearly increase the risk of IHA [11]. A very interesting population study was conducted in Great Britain, which included patients with autoimmune disorders (including IBD), who were administered with GCs. The dosage and the time of administration were investigated, and it was shown that the cumulative dose of GCs has a more significant impact on the IHA risk (in all investigated diseases) than the activity of the underlying disease. Moreover, it also increased at the sustainable dose of prednisolone (<5 mg/day). In the period of one year, the cumulative IHA risk increased from 1.4%—in the periods where GCs were not used—to 8.9% when a prednisolone dose of ≥25 mg/day was administered [52]. Furthermore, it is worth bearing in mind that GCs also affect other risk factors of IHA, such as hyperlipidaemia, hyperglycaemia, and hypertension.

5-aminosalicylates—frequently used in UC—have anti-inflammatory and antiplatelet activity; therefore, they may be preventive in terms of thromboembolism incidents in IBD [11]. TNF-α blockers display strong anti-inflammatory properties; however, there is currently no evidence of their protective effect on the risk of IHA or CV in IBD patients. Although the concentrations of TNF-α are increased among patients with cardiac failure, there is no evidence that anti-TNF- α drugs have a positive influence on the disease, which is still one of the main contraindications of their administration [53,54].

### 3.3. Gut Microbiota in IBD and CVD

The fact that gut microbiota constitutes an integral part of the body has been known for many years, and disorders of its composition—called dysbiosis—are reflected in the form of pathologies of many systems and organs. One of the factors contributing to the inflammatory process in non-specific inflammatory bowel diseases is an abnormal immune response to the disruptions of the intestinal microbiota in the genetically susceptible individuals [55]. On the other hand, the inflammatory process involving the mucous membrane of the intestinal wall directly affects the composition of the microorganisms. This, in turn, can have significant systemic consequences for a particular patient and predispose them to the development of various diseases. One of these conditions may be cardiovascular disease, which, despite the absence of other common factors, such as dyslipidaemia, hypertension or type 2 diabetes, is more common in patients with IBD [56]. Ischaemic heart disease in IBD patients can further deteriorate the quality of life and lead to premature death. Furthermore, patients with non-specific enteritis are at almost three times the risk of thromboembolism, thus, they may experience a thromboembolic incident at a younger age [57].

Many studies have documented significant differences in the gut microbiota composition between patients with non-specific enteritis and healthy individuals, both in terms of the type and the number of microbes. The abundance of intestinal bacteria from the group of *Firmicutes*—especially *Faecalibacterium prausnitzii*—is significantly lower in the faeces of patients with CD. A decrease both in the diversity and the ratio of *firmicutes/bacteroidetes* in the intestinal microbiome is directly associated with a higher incidence of hypertension [58,59,60]. Moreover, hypertension can also be triggered in experimental studies in normotensive rats by replacing the intestinal microbiota between the two strains [61]. Conversely, the amount of bacteria in the Proteobacteria cluster, e.g., *Enterobacteriaceae* including *Escherichia coli*, is often increased in patients with IBD as compared to healthy subjects [62,63]. An increase in the amount of *Enterobacteriaceae* is also observed in persons with CVD, whereas the amount of Streptococcus spp. positively correlates with the diastolic and systolic blood pressure values, thus contributing to an increased risk of cardiovascular disease [64]. Patients with ischemic stroke and transient ischemic attack (TIA) also showed an elevated number of opportunistic bacteria, such as *Enterobacter* and *Oscillibacter,* as well as a decreased number of commensal bacteria, such as *Bacteroides*, *Prevotella* and *Fecalibacterium*. In addition, it is interesting to observe that the severity of dysbiosis correlated with the severity of stroke [65].

The impaired composition of the intestinal microbiome in patients with IBD is also associated with the development of atherosclerosis, as well as with increased markers of arterial stiffness [66]. This is likely to be related to the metabolites secreted by gut bacteria, such as indole and phenyl derivatives [67]. Indole and its derivatives exacerbate the advanced atherosclerotic process of the arteries, while phenyl derivatives of hippuric acid correlate with the occurrence of serious adverse cardiovascular events. In addition, indole has also been found to affect blood pressure by means of peripheral and central mechanisms dependent on serotonin signalling. Finally, indole and indoxyl sulphate can mediate the interaction between gut bacteria and the circulatory system [68].

Another mechanism responsible for increasing the risk of CVD in IBD is the damage to tight intercellular connections due to intestinal dysbiosis, as well as an increase in intestinal permeability. This leads to an increased absorption of endotoxins, particularly lipopolysaccharide (LPS), from the intestines. Subsequently, these endotoxins can enter the systemic circulation, which causes chronic systemic inflammation, including vascular endotheliitis, which intensifies the process of atherogenesis. In addition, a highly inflammatory LPS enhances the synthesis of other pro-inflammatory cytokines, causes LDL oxidation and macrophage activation, as well as increases CRP. The aforementioned factors altogether increase the risk of developing coronary heart disease and, indirectly, of heart failure [69].

In addition, an association between bacterial genes coding for TMA lysis and coronary heart disease has also been observed [64]. The TMA lysis derived from the gut microbiome is involved in the production of N-oxide trimethylamine (TMAO), which plays an important role in the pathogenesis of coronary heart disease [70]. In fact, the use of probiotic-*Bifidobacterium animalis* subsp. LKM512-resulted in a decrease in both TMA and in the number of TMA-producing bacteria, such as Clostridium, the amount of which is usually increased in IBD. Therefore, the use of this probiotic may reduce the risk of atherosclerosis.

Patients with IBD did not show reduced levels of dominant short-chain fatty acids (SCFA)-producing bacteria (e.g., *Faecalibacterium prausnitzii* and *Roseburia intestinalis*) in the mucosa [71]. Studies have shown that SCFA has a modulating effect on the cardiovascular system, and that the administration of acetate may lower blood pressure in experimental models, whereas butyrate supplementation has been found to reduce both systolic and diastolic pressure in T2DM patients [72,73]. Furthermore, the level of formate in the urine strongly correlates with blood pressure [74]. However, taking into account the reduced production of SCFA in IBD patients, their hypotensive effect may be severely limited [75], and the cardioprotective effects of SCFA are associated with the activation of the Gpr41 receptor. Interestingly, a diet with the addition of 1% butyrate reduced the area of aortic damage in mice by up to 50% [76]. In fact, butyrate also reduces the amount of peroxide and nitrotyrosine, which directly reduces oxidative stress and has an anti-atherosclerotic effect [76]. Acetate, propionate and butyrate interact with various G-protein-conjugated receptors [77]. Finally, increasing the number of polyphenols, flavonols, anthocyanins in the diet of IBD patients causes a decrease in inflammation, improves the function of the intestinal microbiota and can have a protective effect on the cardiovascular system [78].

## 4. Dietary Support, Supplementation and Imaging Techniques

Diet in IBD may seem controversial, since dietary guidelines usually suggest a limited intake of dietary fibre, which may further decrease the consumption of fresh fruits and vegetables. Hence, the consumption of simple carbohydrates may be increased, leading to intestinal microbiota dysbiosis and inflammation [79]. On the other hand, a diet rich in dietary fibre and a limited amount of carbohydrates and fats may support IBD remission and decrease inflammation [80]. It should be noted that dietary patterns consistent with the aforementioned diet can also decrease the CV risk [81]. Moreover, dietary guidelines should include the preventive aspects for both CVD and IBD.

### 4.1. Mediterranean Diet

#### 4.1.1. Mediterranean Diet in CVD

**Figure 1:**
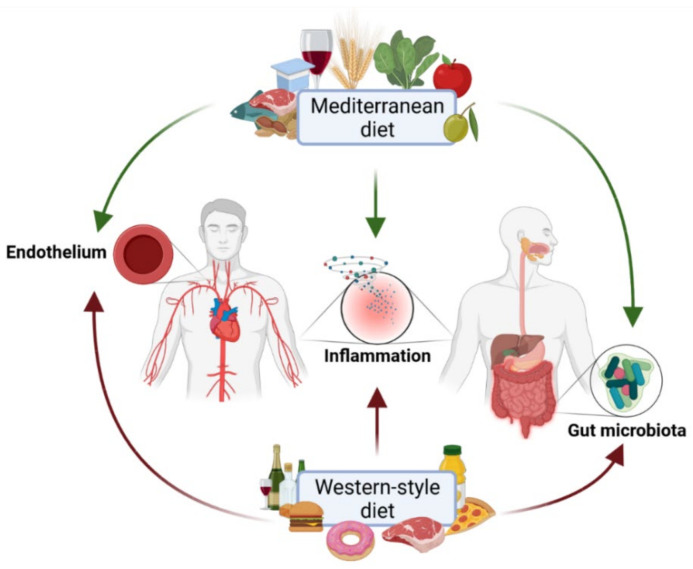
The influence of Western-style and Mediterranean diets on the cardiovascular risk in inflammatory bowel disease.

The Mediterranean diet (MD) is a high-quality, healthy dietary approach in the prevention and treatment of CVD and has been included in the recommendations of several societies as a means to reduce cardiovascular (CV) risk. However, it is worth pointing out that the available evidence is extremely varied, and in several cases, the outcome of the studies should be interpreted with caution [82,83,84,85]. The MD decreases cardiovascular risk factors by affecting the components of the metabolic syndrome, such as positive influence on lipid and glucose profile, body weight, and waist circumference, which improve endothelial functions, lower plasma concentrations of inflammation markers and carotid atherosclerosis (Figure 1) [86,87,88,89,90,91].

#### 4.1.2. Mediterranean Diet in IBD

Adherence to the Mediterranean diet is low among IBD patients. According to Vrdoljak et al., only 9.6% of patients with IBD meet the criteria for its use. Additionally, they also observed low adherence rates regarding the intake of fresh fruit and vegetables [92]. On the other hand, patients suffering from Crohn’s disease in remission presented a greater adherence to the MD diet than patients with an active disease [93]. Moreover, better adherence to a modified Mediterranean diet was associated with a lower risk of Crohn’s disease, although not of ulcerative colitis [94]. In fact, as research suggests, the Mediterranean diet reduced intestinal inflammation in IBD children in clinical remission [95]. It is vital to note that the results of an in vitro and in vivo study revealed that extra virgin olive oil (an important element of the MD diet) might improve disease symptoms in immune-mediated inflammatory diseases [96]. Chicco et al. reported that IBD patients who followed MD diet for six months presented higher BMI and the concentrations of inflammatory biomarkers decreased [97]. Another study demonstrated that following a Mediterranean-inspired anti-inflammatory diet for 6-week reduced inflammation markers and normalised gut microbiota in CD patients [98]. In addition, adherence to the MD diet was associated with a lower calprotectin level among UC patients following pouch surgery [99]. Interestingly, as studies indicate, poor adherence to MD diet constitutes a risk factor of UC [100].

Despite the benefits the MD diet offers in IBD, some of its rules are difficult for patients to follow. For instance, patients suffering from IBD reported an increase in the gastrointestinal symptoms after consumption of fresh vegetable or fruits. Therefore, it seems reasonable to assume the diet is not suitable in every single case and patients should adhere to the MD diet to the best of their tolerance.

### 4.2. Western-Style Diet

#### 4.2.1. Western-Style Diet in CVD

The Western-style diet (WsD) has been shown to be positively correlated with an increased expression of endothelial adhesion molecules (e.g., e-selectin, soluble intercellular adhesion molecule 1, or soluble vascular cell adhesion molecule 1), or higher concentrations of c-reactive protein, associated with an increased risk of CVD, inflammation, and endothelial dysfunction, increasing the risk of such diseases as atherosclerosis [101,102]. Moreover, WsD increases the risk of metabolic disorders, including obesity, type 2 diabetes or the metabolic syndrome, which also constitute significant risk factors for CVD [103,104]. Furthermore, WsD is rich in saturated and trans-fatty acids, which negatively affect the lipid profile, thus, increasing the CV risk.

#### 4.2.2. Western-Style Diet in IBD

The study by Racine et al. indicated that an unbalanced diet, with a high consumption of sugar and soft drinks and a low intake of vegetables, increased the risk of ulcerative colitis [105]. On the other hand, long-term intake of dietary fibre was associated with a decreased risk of CD, but not of UC [106]. Nevertheless, a meta-analysis showed that the Western diet increased the risk of developing both UC and CD [107]. Additionally, an animal study revealed that a diet rich in salt exacerbates inflammatory pathology [108], whereas a high consumption of red meat did not affect the remission duration when compared with a low consumption among patients suffering from CD [109].

The Western diet causes gut microbiota dysbiosis which is an underlying factor in a number of inflammatory diseases, including inflammatory bowel diseases (Figure 1) [110]. A high-fat, high-carbohydrates and high-sugar diet leads to an increase in *Prevotella*, *Bacteroides* and *Escherichia*, which are involved in colitis. On the other hand, a high-fibre diet stimulates the development of *Bifidobacterium*, which induces remission in UC patients [111].

### 4.3. Calcium and Vitamin D in CVD and IBD

Vitamin D is soluble in fat, and can be produced endogenously in the skin following ultraviolet B (UVB) radiation (290–315 nm) [112,113]. However, several food products can also constitute sources of vitamin D, for instance, plants and mushrooms (vitamin D, ergocalciferol), sea fish, fish oil, animal liver, and dairy (vitamin D3, cholecalciferol) [114,115].

Vitamin D deficiency is an issue affecting the entire world [116], although it is the patients with IBD who are a group presenting an increased risk of vitamin D deficiency. This, in turn, results in an increased risk of consequences associated with lower vitamin D concentration levels. The main identified causes include a decreased sun exposure (usually associated with a lower sun exposure, due to the reluctance of patients to leave their homes), an increased photosensitivity associated with pharmacotherapy, inappropriate diet, cigarette smoking, an active phase of the disease, intestinal mucosal inflammation, and their conditions following intestinal resection [117,118]. As a prohormone, vitamin D has to be transformed into the active form [119]. Vitamin D receptor—VDR—is present in many tissues and determines its “classical” function, associated with calcium-phosphate homeostasis and bone metabolism, as well as a pleiotropic function [120]. Vitamin D increases intestinal absorption of calcium and phosphate, increases the tubular reabsorption of calcium in the kidneys, stimulates bone turnover, and synthesises the vitamin D binding protein [121]. A multi-directional activity of vitamin D was confirmed in many observational studies, which highlight the association between lower serum vitamin D concentrations and an increased risk of cancers (colorectal, breast, prostate), multiple sclerosis, type 1 and 2 diabetes, IBD, depression, hypertension, and many of the CVDs [120,122,123]. The association between vitamin D and CVD is essential, as both of these elements affect the majority of the population and are the main causes of an increased morbidity and mortality [124].

Vitamin D can contribute to the development of cardiovascular diseases in many ways by means of modulating the inflammatory response, influencing tissue calcium processes, or regulating the renin-angiotensin-aldosterone (RAA) system [125,126]. Therefore, the potential importance of vitamin D in the context of the development and course of cardiovascular diseases in patients with IBD is considered.

In the Third National Health and Nutrition Examination Survey (NHANES III), a negative correlation was observed between vitamin D concentrations and blood pressure [127,128]. Pathomechanisms of this phenomenon have not been fully described; however, the potential causes comprise disorders of the regulation in the renin-angiotensin-aldosterone (RAA) system and disorders of the endothelial function and smooth muscles of the vessels caused by vitamin D deficiency. At the same time, the inhibitory effect of vitamin D on RAA activity and renin gene expression has been postulated [129]. Therefore, as a risk group of vitamin D deficiency, patients with IBD may also be potentially exposed to the RAA dysregulation. Another important factor leading to hypertension is the imbalance between vasoconstriction and vasodilatation. Wong et al. have shown that vitamin D metabolites can contribute to reduced endothelial spasms of smooth vascular muscle cells [130]. This phenomenon may be particularly important in patients with IBD who are additionally exposed to endothelium cell dysfunction caused by circulating pro-inflammatory cytokines [56].

Atherosclerosis constitutes an important risk factor of CVD, and it is strongly associated with the chronic inflammation in the vessel walls initiated by the disruption of the endothelium. Increased concentrations of lipoproteins, particularly low-density lipoprotein (LDL), lead to their modification and absorption by macrophages, resulting in the formation of foam cells. In turn, vitamin D supplementation leads to a decrease in the accumulation of LDL in macrophages, which causes an inhibition in the formation of atherosclerotic plaque and the development of atherosclerosis. [131]. Moreover, vitamin D can lower the risk of atherosclerosis also by modulating the inflammatory response and decreasing the expression of pro-inflammatory cytokines, such as TNFα, IL-6, IL-1, and IL-8 [132]. It can be especially essential in the IBD population, due to the chronic inflammation and the overproduction of pro-inflammatory cytokines, leading to an increased risk of atherosclerosis. Therefore, apart from its positive impact on the bone structure, vitamin D supplementation may also positively affect the CV risk.

Chronic inflammation among IBD patients can result in the hypercoagulable state and an increased mean platelet volume (MPV), leading to their activation and an elevated risk of thromboembolic events [42]. Vitamin D modulates the expression of thrombomodulin, inhibiting the platelet aggregation and the thrombogenic activity, whereas its deficiency may increase the blood clotting and the risk of embolism.

Vitamin D can also indirectly influence the CV system by affecting the lipid and glucose profiles. In fact, the association between vitamin D deficiency and type 1 and 2 diabetes mellitus (T1DM, T2DM) has been proven [133]. Simultaneously, it should be noted that patients with IBD present an increased risk of carbohydrate-metabolism disorders, due to the administration of glucocorticoids. Furthermore, sun exposure and vitamin D concentrations above 30 ng/mL have been found to be associated with lower concentrations of LDL and higher levels of HDL.

Calcium occurs in the human body mainly in the form of hydroxyapatites in the bones, as well as both an intracellular and extracellular fluid. It participates in muscle contractions, enzymatic activation and signal transmission, as well as in clotting and inflammatory processes. The primary sources of calcium in food are dairy products. Additionally, calcium homeostasis is influenced by parathyroid hormone, calcitonin, and vitamin D. Patients with IBD are at a risk of calcium deficiency; which is due to absorption disorders resulting from the damage to the mucosa of the gastrointestinal tract, as well as from the administrated medications (glucocorticoids), which can increase the excretion of calcium in the urine [134]. Nevertheless, according to a meta-analysis by Bolland et al., the influence of calcium on the CV risk has been debateable, since the researchers showed that calcium supplementation among women increased the risk of heart failure by 27% [135]. However, the abovementioned results have been subject to controversy, and many other publications questioned their methodology and generalisation. Currently, there is evidence that calcium supplementation can be protective against developing CVD. In contrast, in a prospective cohort study conducted by Li et al., calcium supplementation decreased the risk of heart failure by 30% [136]. Moreover, calcium supplementation can positively affect the lipid profile, blood pressure and further decrease the risk of atherosclerosis [137]. Therefore, calcium supplementation and the proper dietary intake is essential in the IBD group.

### 4.4. Imaging Techniques and IBD

As a result of inflammation, the use of classical coronary risk assessments scales (e.g., SCORE) in IBD patients may lead to an underestimation of high-risk patients. Many studies confirmed that additional examinations, such as intima-media thickness measurement, should be performed among IBD patients, since patients are often reclassified into a high cardiovascular risk group in the course of such an assessment. Therefore, a carotid ultrasound examination may be a useful risk assessment tool. According to various studies, patients with IBD have been shown to have an increased carotid intimal thickness and wall stiffness [138,139,140,141]. Ultrasonography variables, such as the carotid intima-media thickness and the presence of carotid plaque, may be helpful to estimate the cardiovascular risk [138,139,140]. Additionally, it may be necessary to perform echocardiography in order to diagnose left ventricular hypertrophy, impaired relaxation or contractility of the left ventricle. It is crucial to note that the potential abnormalities found in echocardiography have an additional predictive power [139]. Hence, it seems that regular monitoring for biomarkers of ischemic heart disease should be considered for an early detection of the disease. In fact, according to American Heart Association (AHA) guidelines in primary prevention, a chronic statin therapy should be applied in patients at a borderline or intermediate risk [142]. Therefore, considering all the aforementioned factors, there is a need to create specific guidelines and risk assessment tools for this specific group of patients.

## 5. Summary and Conclusions

The presented review demonstrates that patients with IBD show an increased risk of CVD in comparison with the general population. At the same time, the typical risk factors for cardiovascular disease in this specific group of patients are not as relevant as in the general population.

Currently, there is a lack of evidence defining cardiovascular disease prevention in patients with IBD. Nevertheless, in order to predict CVD risk, this group of patients should be screened for vascular risk factors, including blood pressure, cholesterol level, smoking, body mass index and the use of a validated CVD risk score. It is essential to remember that certain mechanisms, where IBD increases CVD risk, may not be captured by the clinical risk scores which may potentially lead to underestimating the risk. What is more, SCORE charts have not been validated in adults with IBD. Therefore, it is suggested that cardiovascular risk assessment in patients with IBD using non-invasive imaging techniques, such as the evaluation of atrial stiffness or aortic intima-media thickness (aIMT), should constitute an integral part of IBD treatment, not only following the diagnosis, but also in the course of the patients’ evaluation.

Although the MD diet may be beneficial for IBD patients, some of the rules of this diet may be difficult to implement, e.g., due to the gastrointestinal symptoms after consuming fresh vegetables or fruits. Nevertheless, IBD patients should adhere the MD diet to the best of their tolerance, as it may be beneficial for decreasing CV risk. On the other hand, the consumption of a Western-style diet can increase the risk of UC, CD and CVD; thus, the IBD population should avoid a Western-style diet. Furthermore, both vitamin D and calcium can demonstrate preventive activity in developing CVD among patients with IBD. However, further studies—particularly randomised—are necessary. In fact, the development of specific guidelines for vitamin D and calcium supplementations may be of clinical importance in the IBD group. Moreover, the results of observational studies indicate the need for specific supervision of a cardiovascular disease risk in patients administered with GCSs.

Additionally, it seems that in patients with IBD a proper and effective control of the disease activity is the primary and the most crucial form of CVD prevention. Hence, alleviating inflammation can definitely contribute to reducing the risk of cardiovascular disease in this group of patients.

Finally, the proper choice of medications, diet, vitamin D supplementation, as well as promotion of an active and healthy lifestyle should be emphasised. These elements should be adapted by patients with IBD, as the aforementioned factors are relevant to both reducing the disease activity and decreasing the risk of CVD. Simultaneously, preventive measures, such as early screening and diagnostics, should also be introduced in this patient population.

## Data Availability

Data are available and publicly accessible. The data presented in this study are openly available in the Medline and PubMed databases and on the publisher’s website. The keywords that were used include “inflammatory bowel disease”, “cardiovascular disease”, “Mediterranean diet”, “Western diet”, “inflammation”, “pharmacotherapy”, “glucocorticoids”, “intestinal microbiota”, “intestinal dysbiosis”. All data in the text are quoted and all works used are listed in the bibliography along with doi and reference numbers.
